# Demoralization and Mental Disorders in Community-Dwelling Older Adults: Cluster Analysis

**DOI:** 10.2196/84657

**Published:** 2026-08-19

**Authors:** Alice Onofrio, Maria Ferrara, Ilaria Domenicano, Martino Belvederi Murri, Tommaso Toffanin, Maria Giulia Nanni, Alessandra Canuto, Kerstin Weber, Jana Volkert, Holger Schulz, Martin Härter, Sylke Andreas, Luigi Grassi

**Affiliations:** 1 Department of Neurosciences and Rehabilitation, University of Ferrara, via fossato di Mortara 64/a, Ferrara, 44121, Italy, 39 0532455813, 39 0532212240; 2 Integrated Department of Mental Health and Pathological Addiction, Local Health Trust (AUSL) Ferrara, Ferrara, Italy; 3 Department of Psychiatry, Yale School of Medicine, New Haven, CT, United States; 4 Valais Hospital - Hospital Centre of French-speaking Valais (CHVR), Sion, Switzerland; 5 Department of Psychiatry, University of Geneva, Geneva, Switzerland; 6 Clinic for Psychosomatic Medicine and Psychotherapy, University of Ulm, Ulm, Germany; 7 Department of Medical Psychology, University Medical Center Hamburg-Eppendorf, Hamburg, Germany; 8 Institute of Psychology, Alpen-Adria University Klagenfurt, Klagenfurt, Austria

**Keywords:** demoralization, depression, anxiety, cluster, older

## Abstract

**Background:**

Demoralization negatively affects the quality of life, especially in people with mental disorders.

**Objective:**

This study aims to assess demoralization in a cohort of community-dwelling older adults, to identify groups of individuals who share similar characteristics in terms of mental health conditions and the severity of demoralization domains, and to investigate the different characteristics across these groups.

**Methods:**

Participants were enrolled in 3 centers as part of the cross-sectional MentDis_ICF65+ study and were assessed using the Demoralization Scale and the Composite International Diagnostic Interview at baseline. A cluster analysis was conducted using the partitioning around medoids algorithm with Gower’s distance to classify individuals based on demoralization severity and the diagnosis of mental health disorders, allocating individuals to a distinct number of patterns by examining their similarities across these variables.

**Results:**

Among the final sample of 1369 participants, 3 clusters were identified: the “low psychopathology” cluster, with very few mental health diagnoses; the “affective” cluster, characterized by affective disorders (100% of participants); and the “anxiety” cluster, characterized by anxiety disorders (100% of participants). Men were prevalent in the “low psychopathology” cluster (n=635, 59.1%; *P*<.001), while women were more prevalent in the “affective” cluster (n=133, 72.3%; *P*<.001) and the “anxiety” cluster (n=79, 71.8%; *P*<.001). The “affective” cluster had the highest mean scores for disheartenment (17.1, SD 6.7; *P*<.001), dysphoria (12.1, SD 3.5; *P*<.001), and loss of meaning (11.8, SD 4.9; *P*<.001) but the lowest for sense of failure (17.4, SD 3.2; *P*<.001).

**Conclusions:**

The identification of different clusters of demoralization and mental health disorders in community-dwelling older adults, as well as sex-based differences, highlights the need for tailored mental health interventions in this vulnerable population.

## Introduction

Demoralization was conceptualized more than 40 years ago as a state of existential distress denoting a persistent failure to cope with stress and occurs in patients with severe physical or mental illnesses, specifically those that threaten life or the integrity of being [1]. Subsequently, it has been described as a syndrome characterized by loss of purpose and meaning, a state of persistent feeling of subjective incompetence and existential distress, hopelessness, and helplessness, and an inability to cope and solve problems [2-4]. As a construct, demoralization only partly overlaps with depression, with demoralized individuals not experiencing the lack of motivation typical of major depression, but rather an uncertainty in the appropriate course of action that results in an inability to cope with external sources of distress [5-7].

Demoralization has been extensively investigated in patients with medical comorbidities, including, but not limited to, those with cardiac, dermatological, and oncological conditions [8-10], as well as mental disorders. Additionally, demoralization has been shown to be associated with suicidal ideation, poorer quality of life, and worse outcomes in both mental and somatic disorders [7,11,12]. It has also been established that the prevalence of mental health diagnoses increases with demoralization [13,14] and that demoralization might play a role in the development or exacerbation of mental health conditions, such as anxiety disorders or depression [15].

In the older population, demoralization syndrome has not been examined in detail, apart from a few authors, such as Ramm et al [16], who found a higher prevalence of demoralization in people aged ≥65 years compared to younger people, as well as an increase in older adulthood (65-74 y, 75-84 y, and 85 yrs and older, 50% prevalence).

Since previous studies among the older individuals with mental disorders, especially depression, showed that they had a worse quality of life and higher levels of disability compared to their peers without a mental health diagnosis [17], it is imperative to understand the impact of demoralization in the aging population [18].

By using cluster analysis, or clustering, a machine learning technique that can automatically identify natural groupings in data by decomposing heterogeneous data [19], the impact of demoralization and mental health disorders in the older population could be examined in a proper way. While predictive modeling is used to predict the already-existing class, clustering algorithms play a key role when the classes are unknown, and the instances have to be classified into [20]. Cluster analysis offers a better understanding of the heterogeneity that exists in patient characteristics in clinical populations [21]. This method has already been applied to identify subtypes of medical patients suffering from demoralization. For instance, Rafanelli et al [22], by studying 1560 medically ill patients, found that 373 had demoralization, and 4 clusters were identified: in 3 out of 4 clusters, demoralization was comorbid with a mental health diagnosis (either major depression, anxiety, and somatoform disorders); in one cluster, it was not associated with any mental health condition (110/373, 29.5% of the sample). Cluster analysis has also been successfully applied to a large mental health registry to describe subgroups of patients in order to program resource allocations [23].

The aims of this study were to assess demoralization in a cohort of community-dwelling older adults and to identify whether groups of individuals cluster together by sharing similar characteristics in terms of demoralization scores as well as domains and mental health disorders. A secondary aim was to investigate clinical and sociodemographic differences across those groups.

## Methods

### Design

We examined data from the European project Mental Disorders in the Elderly (MentDis_ICF65+), a multicenter study aimed at investigating mental disorders in the community-dwelling older population, including incidence, prevalence, and correlations with the intensity of symptoms, levels of engagement in activities, participation, and use of services [24,25].

The study was conducted in 6 countries (Germany, Great Britain, Israel, Italy, Spain, and Switzerland) and included 3142 participants aged between 65 and 84 years of age. The exclusion criteria were severe cognitive impairment and insufficient proficiency in the corresponding language. It consisted of a community survey administered at baseline, with a follow-up at 12 months. The survey aimed to collect 12-month and 1-month prevalence and comorbidity of mental disorders, as well as information on the prior course of the disorders, quality of life, health care use, help-seeking behavior, impairments, and participation in community life.

Only 3 centers (ie, Ferrara, Geneva, and Hamburg) out of the 6 centers examined the construct of demoralization in addition to the standard protocol and were, therefore, included in the present analysis.

### Measures

All participants in the 6 centers underwent individual clinical interviews, along with the completion of psychometric instruments. Additionally, aspects such as quality of life, health care use, help-seeking behavior, impairments, and participation in community life were assessed.

Mental health was assessed using the Composite International Diagnostic Interview for the Elderly (CIDI65+) [26], a version of the Composite International Diagnostic Interview (CIDI) specifically adapted to the needs and abilities of the older individuals. The CIDI65+ test-retest reliability has been examined and was considered satisfactory [26]. This interview assessed the 1-month and 12-month prevalence of somatic morbidity, somatoform disorders, anxiety disorders, obsessive-compulsive disorders, affective disorders, psychotic symptoms, substance abuse, adjustment and stress-related disorders, and cognitive impairment. For the purpose of this investigation, only the 1-month prevalence was considered.

The assessment of physical health included a checklist of primary medical conditions (such as cardiovascular disease, cancer, and musculoskeletal disease) self-reported by the participants, as well as a survey on the use of medications and access to health care facilities. Demographic and socioeconomic data were also gathered as part of the standard assessment.

The Demoralization Scale (DS), developed by Kissane et al [27], is a 24-item self-report tool measuring demoralization syndrome through a 5-point Likert scale (“never”=0; “all the time”=4) in 4 domains: disheartenment, loss of meaning or purpose, dysphoria, and sense of failure. The disheartenment domain includes feelings of discouragement and isolation, the loss of meaning domain pertains to the loss of role, purpose, and sense of worth in life; the dysphoria domain encompasses emotions of distress and regret; and the sense of failure domain assesses the levels of accomplishment experienced by the individual [27]. The overall demoralization score (DS-Total) is calculated by summing item scores, ranging from 0 to 96. A total score equal to or exceeding 30 is indicative of a demoralized status, according to the criteria established by Kissane et al [27]. The DS has previously demonstrated robust levels of validity and reliability in several populations, such as patients with cancer [28], medically ill patients [5], and patients with nonpsychotic affective disorders [29].

### Statistical Analysis

Descriptive statistics were computed for all study variables. Individuals were classified as demoralized in a categorical variable, according to Kissane [27].

In this study, we applied a cluster analysis method to allocate individuals to a distinct number of patterns by investigating individuals’ similarities regarding variables that described several aspects of demoralization and the presence or absence of mental health disorders. Cluster analysis is an exploratory method for identifying groups of objects that share common characteristics. Generally, the groups are built such that objects belonging to the same groups share a similar response pattern on the observed variables, while such patterns are as different as possible between objects in different groups [30].

Eight variables were used as classifiers, namely (1) four continuous variables aimed at describing the severity of demoralization, that is, four domains of the DS as identified by Grassi et al [8]: disheartenment, sense of failure, dysphoria, and loss of meaning and purpose; (2) four binary variables aimed at identifying the presence or absence of mental health disorders: anxiety disorders (phobic disorder, panic attack, panic disorder, generalized anxiety disorder (GAD), obsessive-compulsive disorder), affective disorders, and nonaffective psychosis (major depressive disorder, dysthymic disorder with or without hierarchy, double depression, bipolar 1, bipolar 2, psychosis), alcohol dependence or abuse disorder, and somatoform pain disorder. Individuals with missing information regarding mental health diagnoses (5/1479) or demoralization (105/1479) were excluded from the cluster analysis.

Raw scores on demoralization domains were standardized to z-scale scores based on their mean and SD in the full sample pooled across centers to prevent variables with larger numerical ranges from dominating the clustering procedure [31]. Since the data contained both continuous and categorical variables, Gower’s similarity measure was used to generate the matrix of similarities [32]. In computing the similarity measure, the same weight was assigned to each variable, and binary variables were treated as nominal variables. Based on this matrix, individuals were clustered by the partitioning around medoids (PAM) algorithm [33]. For a given number of groups, PAM allocates individuals so that the dissimilarity to the medoid is minimized within groups. We used several criteria to select the optimal number of clusters over 2‐7 cluster solutions. First, we constructed a total within sum of squares (WSS) plot and used the so-called elbow method to identify the optimal number of clusters so that adding another cluster would not improve the total WSS. Second, we computed the silhouette and identified the number of clusters for which the average silhouette width was maximized. Similarly, we employed the GAP statistics and found the solution cluster that maximized the statistics. Finally, we computed the average distance to cluster centroid to determine which solutions had the lowest value [33]. After identifying the optimal number of clusters, cluster-wise stability was assessed using bootstrap resampling with Jaccard similarity. The Jaccard index, computed as the mean over the Jaccard similarities between the original and the bootstrapped clusters, was used to evaluate the stability of a cluster. An index approaching 1 indicates a stable cluster, while lower values suggest instability [34]. The number of resampling was set to 1000.

Finally, we used t-distributed stochastic neighbor embedding (t-SNE) to visualize them. t-SNE is a nonlinear dimensionality reduction technique that allows the visualization of high-dimensional datasets in a lower-dimensional space. Data can, therefore, be represented as a scatter plot, where points that were close in the original space remain close, and points that were far away in the original space remain out to be far away [35]. The t-SNE representation was used only for visualization purposes, and not as evidence of separability.

After identifying the groups, we assessed the characteristics of the clusters by comparing the variables used for clustering (eg, the 4 subscales of the DS and the presence or absence of mental health disorders).

Furthermore, we assessed the characteristics of the clusters by comparing the external variables (eg, those not used for clustering, including sociodemographic variables such as sex, age, marital status, household, number of school years, working status, perceived financial situation, and physical illness). Differences in the aforementioned sample characteristics were compared according to cluster membership using independent sample ANOVA and *χ*^2^ tests for continuous and categorical variables, respectively. Due to the exploratory nature of the study, analyses were not controlled for multiple testing.

The analyses were conducted with the R software (version 4.3.1; R Foundation for Statistical Computing) [36]. The packages *cluster* [37] and *fpc* [38] were used to perform and evaluate the cluster analysis. For all tests performed, the significance level was set at 0.05.

### Ethical Considerations

The authors assert that all procedures contributing to this work comply with the ethical standards of the relevant national and institutional committees on human experimentation and with the Helsinki Declaration of 1975, as revised in 2008. The study was approved by research ethics committees in all 6 centers (Germany: Hamburg Ethics Committee of the Medical Association No. 2895, Italy: Ferrara No. 0096637 5/11/2009, Israel: Jerusalem No. 0376‐09 -HMO, Spain: Madrid No. 22032010, Switzerland: University Hospitals of Geneva Ethics Committee, Protocol No. 09‐121 and UK: National Research Ethics Service No. 10/H0715/21).

## Results

**Figure 1.**
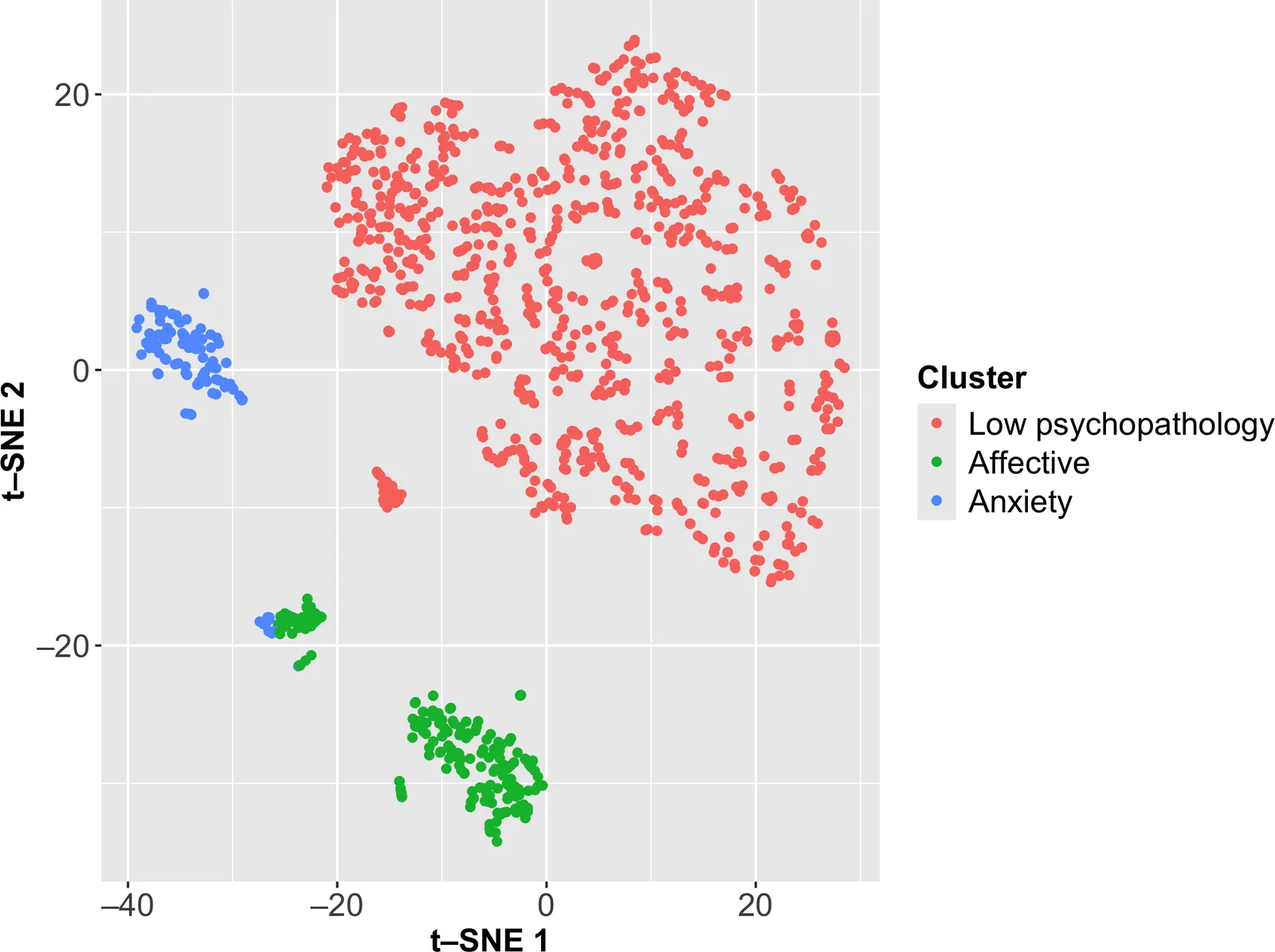
Clusters, as detected by the partitioning around medoids (PAM) algorithm, represented with t-distributed stochastic neighborhood embedding (t-SNE). T-SNE parameters were set as follows: perplexity=30, learning rate=200, iteration=1000, seed=123.

A total of 1479 participants were included in this study. However, 110 were excluded because the information regarding mental health diagnosis (n=5) or demoralization (n=105) was not available, leaving a final sample of 1369 individuals (Ferrara, n=466 participants; Geneva, n=509 participants; and Hamburg, n=394 participants; Figure S1 and Table S1 in Multimedia Appendix 1). Of the 110 missing outcomes, 50.9% (n=56) were female and 49.1% (n=54) were male; 26.4% (n=29) were aged 65‐69 years, 29.1% (n=32) were aged 70‐74 years, 29.1% (n=32) were aged 75‐79 years, and 15.5% (n=17) were aged 80 years or older. Sociodemographic data are summarized in Table 1, while Figure 1 shows a visual representation of the 3 clusters.

**Table 1.** Sociodemographic characteristics of the 3 clusters.

Characteristics	Total (N=1369)	“Low psychopathology” cluster (n=1075)	“Affective” cluster (n=184)	“Anxiety” cluster (n=110)	*χ*^2^ (*df*)	*F* value (*df*)	Cohen^a^ effect size	*P* value
Center, n (%)					34.69 (4)		0.16	<.001
Ferrara	466 (34.0)	383 (35.6)	46 (25.0)	37 (33.6)				
Geneva	509 (37.2)	368 (34.2)	104 (56.5)	37 (33.6)				
Hamburg	394 (28.8)	324 (30.2)	34 (18.5)	36 (32.8)				
Sex, male, n (%)	717 (52.4)	635 (59.1)	51 (27.7)	31 (28.2)	89.98 (2)		0.26	<.001
Age, mean (SD)	73.1 (5.3)	73.2 (5.3)	72.4 (5.3)	72.8 (5.0)		1.75 (2, 1366)	0.05	.17
Age group (y), n (%)					8.47 (6)		0.08	.20
65‐69	435 (31.8)	331 (30.8)	72 (39.1)	32 (29.1)				
70‐74	390 (28.5)	310 (28.8)	43 (23.4)	37 (33.6)				
75‐79	343 (25.0)	268 (24.9)	47 (25.5)	28 (25.5)				
>80	201 (14.7)	166 (15.5)	22 (12.0)	13 (11.8)				
Marital status, n (%)					48.36 (4)		0.19	<.001
Married	865 (63.2)	725 (67.4)	78 (42.4)	62 (56.4)				
Separated/divorced/widowed	439 (32.1)	299 (27.8)	96 (52.2)	44 (40.0)				
Never been married/other	64 (4.7)	50 (4.7)	10 (5.4)	4 (3.6)				
Missing	1 (0.1)	1 (0.1)	0 (0)	0 (0)				
Household, n (%)					36.64 (2)		0.16	<.001
Lives alone	405 (29.6)	278 (25.9)	86 (46.7)	41 (37.3)				
Other	961 (70.2)	796 (74.0)	98 (53.3)	67 (60.9)				
Missing	3 (0.2)	1 (0.1)	0 (0.0)	2 (1.8)				
Number of school years, mean (SD)	11.6 (4.6)	11.7 (4.7)	11.9 (4.7)	10.6 (3.8)		3.36 (2, 1363)	0.07	.04
Number of school years group, n (%)					12.46 (4)		0.10	.01
0‐8	356 (26.0)	278 (25.9)	45 (24.5)	33 (30.0)				
9‐12	469 (34.3)	350 (32.7)	70 (38.0)	49 (44.5)				
13 and more	541 (39.5)	444 (41.4)	69 (37.5)	28 (25.5)				
Working status, n (%)					12.08 (8)		0.09	.15
Working or employed	72 (5.3)	58 (5.4)	8 (4.4)	6 (5.5)				
Homemaker or housewife	34 (2.5)	25 (2.3)	8 (4.4)	1 (0.9)				
Unemployed	1 (0.1)	0 (0)	1 (0.5)	0 (0)				
Retired	1242 (90.7)	975 (90.7)	166 (90.2)	101 (91.8)				
Other	5 (0.4)	4 (0.4)	0 (0.0)	1 (0.9)				
Missing	15 (1.1)	13 (1.2)	1 (0.5)	1 (0.9)				
Financial situation perceived, n (%)					21.40 (8)		0.12	.006
Very good	180 (13.1)	144 (13.4)	20 (10.9)	16 (14.5)				
Good	661 (48.3)	507 (47.2)	105 (57.0)	49 (44.6)				
Just enough	450 (32.9)	371 (34.5)	41 (22.3)	38 (34.5)				
Poor	63 (4.6)	42 (3.9)	16 (8.7)	5 (4.6)				
Very poor	11 (0.8)	7 (0.6)	2 (1.1)	2 (1.8)				
Missing	4 (0.3)	4 (0.4)	0 (0)	0 (0)				

a Effect sizes are reported as Cohen d (continuous variables) or Cohen w (categorical variables), as appropriate

A total of 317/1369 (23.15%) patients were classified as demoralized. Amongst demoralized individuals, 147 (46%) had a comorbid psychiatric diagnosis. Amongst those who did not have any mental disorder (1041/1369), 16% (n=170) were classified as demoralized (Table 2).

**Table 2.** Distribution of mental disorders and demoralization in the study sample for the cluster analysis (N=1369).

Characteristics	Demoralization score <30, not demoralized (n=1052), n (%)	Demoralization score ≥30, demoralized (n=317), n (%)	Total, n
Mental health disorders			
Anxiety disorders	83 (7.9)	65 (20.5)	148
Affective disorders	83 (7.8)	113 (35.6)	196
Alcohol abuse	28 (2.7)	4 (1.3)	32
Somatoform pain disorders	9 (0.9)	17 (5.4)	26
No mental health disorders	871 (82.8)	170 (53.6)	1041

**Figure 2.**
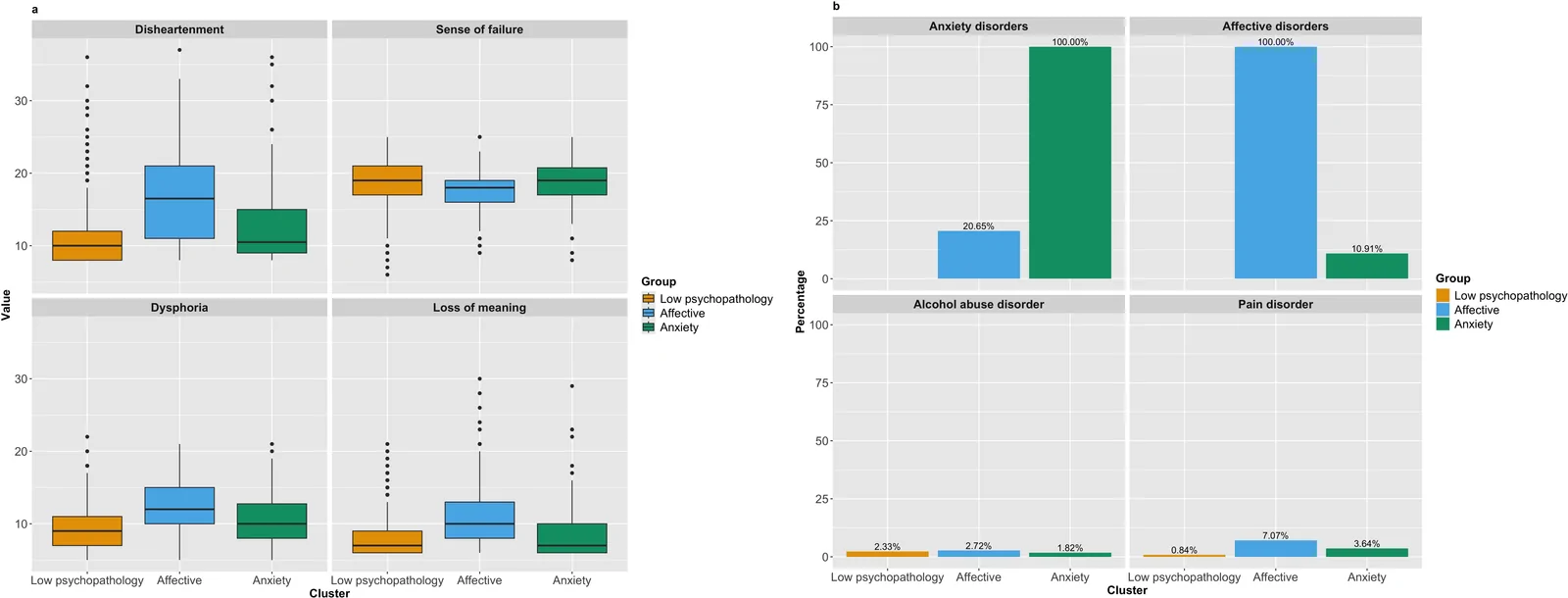
Characteristics of the 3 clusters across the variables used as classifiers (a): demoralization domains; (b): mental health disorders).

The number of clusters that showed the best performance was 3, as it had an adequate average silhouette and elbow shape (Table S2, Table S3, and Figure S2 in Multimedia Appendix 1). The first cluster was characterized by “low psychopathology” (0% anxiety disorders, 0% affective disorders, 2.3% alcohol abuse disorder, and 0.8% somatoform pain disorder); the second was characterized by the presence of affective disorders (100% of participants); and the third by anxiety disorders (100% of participants; Figure 2B). Some individuals had both anxiety and affective disorders: 38 (20.7%) participants in the “affective” cluster also had an anxiety disorder, and 12 (10.9%) participants in the “anxiety” cluster also had an affective disorder (Table 3). All 3 clusters showed a robust structure, as they rarely separated or merged with the others upon resampling: specifically, the Jaccard bootstrap mean was 0.82 for the “low psychopathology” cluster, 0.88 for the “affective” cluster, and 0.83 for the “anxiety” cluster.

**Table 3.** Characteristics of the 3 clusters across the variables used as classifiers (demoralization domains and mental health disorders).

	“Low psychopathology” cluster (n=1075)	“Affective” cluster (n=184)	“Anxiety” cluster (n=110)	*χ*^2^ (*df*)	*F* value (*df*)	Effect size Cohen^a^ (95% CI)	*P* value
Demoralization, mean (SD)							
Total score	22.8 (7.4)	34.1 (11.8)	26.5 (10.9)		141 (2, 1366)	0.45 (0.0-0.50)	<.001
Disheartenment	11.1 (3.7)	17.1 (6.7)	13.0 (6.0)		146 (2, 1366)	0.46 (0.0-0.51)	<.001
Sense of failure	19.0 (3.0)	17.4 (3.2)	18.4 (3.2)		21.47 (2, 1366)	0.18 (0.0-0.22)	<.001
Dysphoria	8.9 (2.8)	12.1 (3.5)	10.5 (3.8)		97.71 (2, 1366)	0.38 (0.0-0.42)	<.001
Loss of meaning	7.9 (2.6)	11.8 (4.9)	8.6 (4.1)		95.14 (2, 1366)	0.37 (0.00.42)	<.001
DS ≥30, n (**%**)	175 (16.3)	112 (60.9)	30 (27.3)	176.7 (2)		0.36 (0.30-0.42)	<.001
Mental health disorders, yes, n (%)							
Anxiety disorders	0 (0.0)	38 (20.7)	110 (100)	1056.3 (2)		0.88 (0.85-0.90)	<.001
Affective disorders	0 (0.0)	184 (100)	12 (10.9)	1281.8 (2)		0.97 (0.95-0.98)	<.001
Alcohol abuse disorder	25 (2.3)	5 (2.7)	2 (1.8)	0.25 (2)		0.01 (0.00-0.07)	.88
Somatoform pain disorder	9 (0.8)	13 (7.1)	4 (3.6)	34.6 (2)		0.16 (0.09-0.23)	<.001
Anxiety disorders + affective disorders	0 (0.0)	38 (20.7)	12 (10.9)	208.3 (2)		0.39 (0.34-0.45)	<.001
Alcohol abuse disorder	0 (0.0)	0 (0.0)	0 (0.0)	—^b^	—	—	—
Somatoform pain disorder	—	4 (10.5)	1 (8.3)	—	—	—	—

a Effect sizes are reported as Cohen d (continuous variables) or Cohen w (categorical variables), as appropriate.

b Not available.

Men were more prevalent in the “low psychopathology” cluster (635/1075, 59.1%; *P*<.001), while women were largely prevalent in the “affective” cluster (133/184, 72.3%; *P*<.001) and “anxiety” cluster (79/110, 71.8%; *P*<.001). There was no significant difference in age between clusters (*P*=.20). A difference in marital status was observed between the 3 clusters: in the “low psychopathology” cluster and the “anxiety” cluster, married people were more prevalent (725/1075, 67.4% and 62/110, 56.4%, respectively; *P*<.001), while in the “affective” cluster, separated or widowed or divorced participants were more prevalent (96/184, 52.2%; *P*<.001; Table 1).

**Figure 3.**
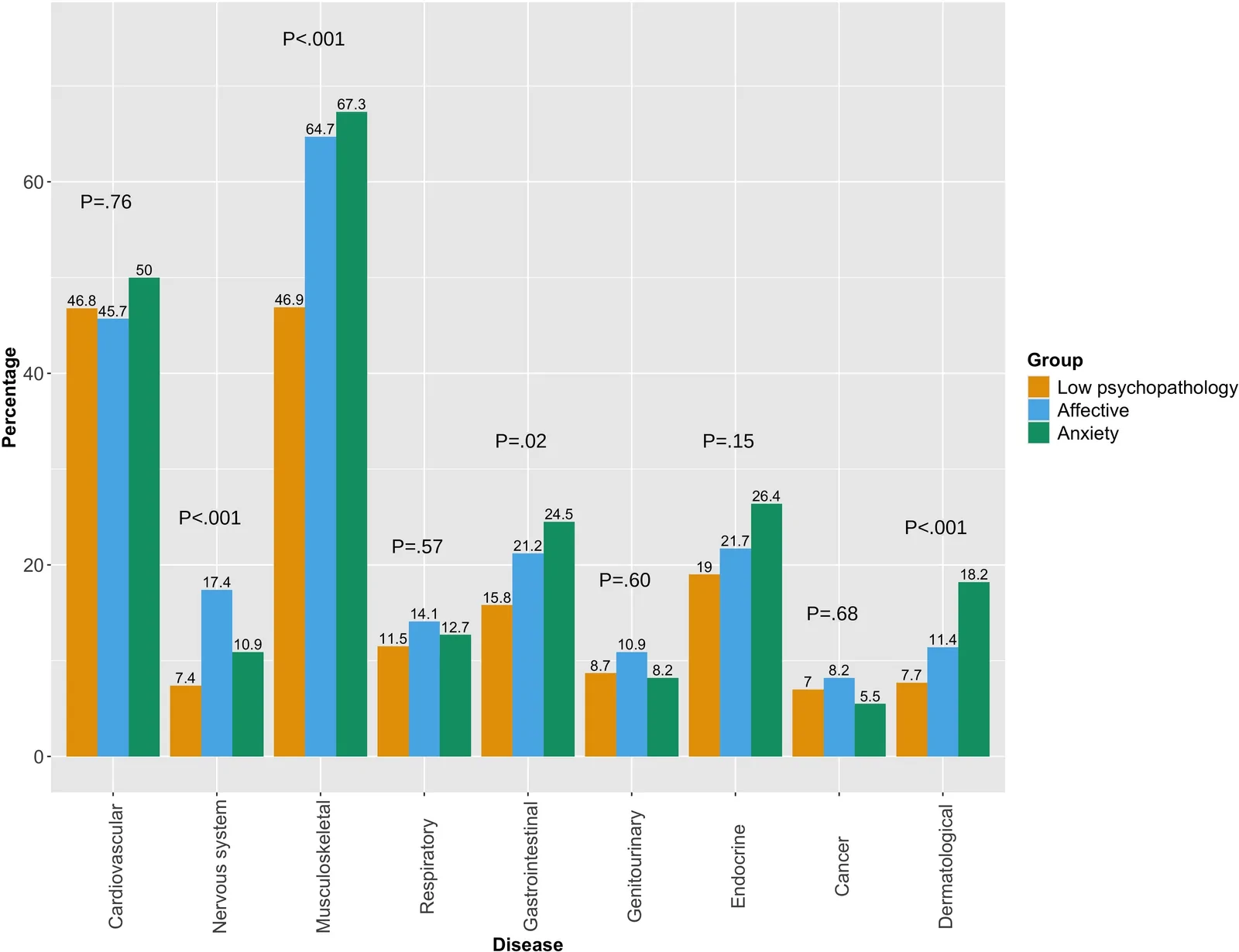
Physical comorbidities across the 3 clusters.

The proportion of people living alone was the highest in the “affective” cluster (n=86, 46.7%; *P*<.001), followed by the “anxiety” cluster (n=41, 37.3%; *P*<.001) and the “low psychopathology” cluster (n=278, 25.9%; *P*<.001). As shown in Figure 3 and Table S4 in Multimedia Appendix 1, the “affective” cluster had the highest prevalence of nervous system (*P*<.001) physical comorbidities, while the “anxiety” cluster had the highest prevalence of musculoskeletal (*P*<.001), gastrointestinal (*P*=.02), endocrine (*P*=.15), and dermatological (*P*<.001) comorbidities.

Regarding the different domains of demoralization, the “low psychopathology” cluster had the lowest scores for disheartenment (mean 11.1, SD 3.7; *P*<.001), dysphoria (mean 8.9, SD 2.8; *P*<.001) and loss of meaning (mean 7.9, SD 2.6; *P*<.001) subdomains, but the highest score for the sense of failure domain (mean 19.0, SD 3; *P*<.001). The “affective” cluster had the highest mean score for disheartenment (mean 17.1, SD 6.7; *P*<.001), dysphoria (mean 12.1, SD 3.5; *P*<.001) and loss of meaning (mean 11.8, SD 4.9; *P*<.001), but the lowest mean score for sense of failure (mean 17.4, SD 3.2; *P*<.001; Table 3).

## Discussion

This study aimed to identify possible clusters based on mental health disorders and demoralization in a community-dwelling older population across 3 European centers. Using the criteria of the methods described above, we identified 3 distinct patterns. The first group was characterized by low psychopathology, the second by the presence of affective disorders, and the third by the presence of anxiety disorders.

About one fourth (23.2%) of the community-dwelling older individuals in our sample were found to be demoralized. This result is very close to the percentage of demoralized participants previously found in medically ill patients of any age [39,40] and in a more recent study of the older population [16]. This result underlines the need to take demoralization into careful consideration, since it is well known that with age, the burden of medical illnesses increases and that medical illnesses are often associated with demoralization [8,10,11,13].

Some participants had both anxiety and affective disorders. The cluster analysis assigned these comorbid patients either to the “anxiety” cluster or to the “affective” cluster based on the other classifiers included in the analysis, that is, the 4 domains of the DS identified by Grassi et al [7,28]. This finding suggests that the assessment of demoralization can help clarify the characteristics of comorbid mental disorders. This could allow the implementation of tailored interventions in otherwise mixed diagnostic categories.

The “affective” cluster showed higher scores in most of the demoralization domains, for instance, higher disheartenment, dysphoria, and loss of meaning. Affective disorders, especially depression, and demoralization syndrome have some overlapping symptoms, such as suicidal ideation, but are, in fact, separate concepts. Demoralization has been defined as a combination of distress and subjective incompetence, without the lack of motivation that characterizes Major Depressive Disorder [3]. Demoralization and Major Depression, however, are often correlated [5,7]. There might be a two-way relationship: depression, as with any other medical condition, could result in demoralization, but at the same time demoralization could precede depression [22,41].

Women were more prevalent in the “affective” and “anxiety” clusters, which confirms gender differences in the prevalence of mental disorders. Mental disorders are more prevalent in females than in males, especially anxiety and mood disorders [42]. The underlying mechanisms of this difference remain unclear and may include society-mediated factors, genetics and hormonal fluctuations, the latter especially in the perimenopausal and postmenopausal period [43].

The “low psychopathology” cluster had the lowest levels of disheartenment, dysphoria and loss of meaning, but the highest sense of failure. The presence of a higher sense of failure in this group, especially compared to the relatively low prevalence in the “affective” cluster, has never been reported before. This finding, which could appear contradictory at first, aligns with the concept of *subjective incompetence* [44,45]. In fact, the main source of distress for older adults without an identifiable psychiatric diagnosis may arise from the comparison between the ever-present need for autonomy and age-related functional limitations, which in turn could trigger a more pronounced reactive perception of personal incompetence. This suggests that demoralization and mental health conditions do not necessarily overlap, corroborating the phenomenological difference between demoralization syndrome and mental health disorders such as depression. Furthermore, the elevation in the “Sense of Failure” domain suggests that, in this cluster, demoralization may act as a dimension of existential distress rather than a marker of a mental disorder ([5]M. Belvederi Murri et al., 2020 [3]. As individuals age, their temporal perspective shifts, often leading them to reflect more on past achievements rather than future goals. In this context, the ’Sense of Failure’ may capture the appraisal of one’s life trajectory rather than acute emotional distress. When this retrospective recollection collides with changes in social roles or functional decline, it can manifest as perceived inadequacy or unfulfilled potential.

The “affective” cluster had a higher prevalence of people living alone, as well as those who were separated, widowed, or divorced. This finding suggests that loneliness plays a role in affective disorders, particularly among older individuals. Previous literature studied this association, but further research may be needed to assess the impact of loneliness on demoralization [46], as also recently indicated in a study of older patients on hemodialysis [47] as well as in older patients with stroke and disability [48].

Nervous system diseases were most prevalent in the “affective” cluster, while musculoskeletal, gastric, endocrinological, and dermatological diseases were most prevalent in the “anxiety” cluster. These findings support the notion that psychopathology correlates with specific physical comorbidities. The correlation between medical conditions and a higher prevalence of mental health diagnoses is well documented in the literature. For example, patients with neurological conditions such as migraines or epilepsy have a higher prevalence of affective disorders compared to the general population [49,50], while anxiety disorders are often associated with gastrointestinal conditions (ie, peptic ulcers or IBS), endocrinological conditions (ie, Graves’ disease), and dermatological diseases (ie, psoriasis) [51,52].

To our knowledge, this is the first study to apply a cluster analysis method to examine the relationship between demoralization and mental health disorders in a sample of community-dwelling older participants. Previous studies have mostly focused on demoralization in medical settings, whereas this study assesses on assessing demoralization among older people in the general population. In addition, the cluster analysis method offers a new strategy for understanding the heterogeneity of participants’ characteristics within the sample. The identification of different clusters in the presentation of mental health conditions provides the opportunity to design more tailored psychotherapeutic, psychoeducational, and psychosocial interventions for the examined population. For the “affective” cluster, psychotherapeutic interventions tailored to older adults may reduce the psychological distress associated with demoralization. For instance, Managing Cancer and Living Meaningfully (CALM), a psychotherapeutic intervention designed to address end-of-life distress in cancer patients [53], could be adapted to accommodate the care needs of older adults, who share a similar burden of disability and loss of meaning. For the “anxiety” cluster, age-adapted psychotherapy and psychoeducation focusing on emotional regulation and coping strategies may be beneficial in reducing anxiety symptoms.

Some limitations need to be acknowledged. First, due to the cross-sectional design of this investigation, causal inferences are not possible. Second, the geographic limitations of the catchment area, as well as the exclusion of individuals with cognitive impairment and others living in residential facilities, those who are homeless, or those with a language barrier might limit the generalizability of the findings. Third, as in most studies on demoralization, we also used a psychometric tool, while more specific data might have emerged through a clinometric and interview-based approach [54,55], which represent the gold-standard for assessing demoralization. In relation to this, a fourth limitation is that we considered a clinical condition indicating demoralization syndrome by using a standard cut-off score of ≥30, which is, in fact, what we found in a global population of medically ill patients. Studies comparing the demoralization interview vs the DS in older individuals are necessary to define a cut-off that optimizes sensitivity and specificity for this construct. Finally, while the DS has been widely adopted in research and clinical contexts, however, its validation is not yet available in the French language.

As reported, 7% of participants were excluded from the analysis due to missing information on diagnosis or demoralization subscales. Since a relatively small fraction of the data was missing, and the dataset presented an adequate sample size, a complete-case analysis was used to handle missing data [56]. However, a sensitivity analysis to compare the current results (obtained through a complete-case analysis) to an imputed dataset would strengthen the study and ensure the robustness of the findings.

A further limitation that must be acknowledged is that psychiatric diagnosis variables were included as binary variables within the clustering features; thus, the clustering algorithm might have been strongly incentivized to separate groups by such variables, potentially dominating the structure and reducing the added value of demoralization dimensions. To address this specific aspect, a sensitivity analysis clustering only on demoralization domains could be performed, subsequently comparing diagnoses across resulting clusters, or focusing on variable contribution by observing changes in silhouette scores when iteratively removing variables. Moreover, binary variables were automatically treated as symmetrical, even if potentially unbalanced. Finally, given the exploratory nature of this study, we did not systematically correct for multiple comparisons, which might increase the risk of Type I errors. To mitigate this issue, effect sizes and confidence intervals were reported.

This study identified three different clusters based on the presentation of demoralization and mental health disorders in community-dwelling older individuals, underlining sex-based differences in the presentation of demoralization and specific dimensions of demoralization, mostly associated with certain mental disorders. Further research is needed to guide tailored mental health interventions to address the vulnerabilities of these specific categories in the older population and the role of demoralization in influencing individuals’ quality of life [57].

## Supplementary material

10.2196/84657 Multimedia Appendix 1 Participant flow diagram; exact missing counts; criteria used to determine the optimal number of clusters; performance of the k-prototypes algorithm; hierarchical clustering with Gower+Ward; physical comorbidities across the 3 clusters; and comparison of baseline characteristics across the 3 centers.
