# Supplementary material for: Demoralization and Mental Disorders in Community-Dwelling Older Adults: Cluster Analysis
Source: JMIR Aging. 2026 Aug 19;9:e84657. doi: 10.2196/84657 (PMC13488920; doi:10.2196/84657)
Supplement: Supplementary file 1 [file aging-v9-e84657-s001.docx]

**Supplementary materials**

**Figure S1. Participants flow diagram**

**Table S1. Exact missing counts by variable and by center.**

|  | Tot | Ferrara | Gen | Hamburg |
| --- | --- | --- | --- | --- |
| demoralization | 105 | 31 | 6 | 68 |
| MH diagnosis | 5 | 3 | 1 | 1 |

**Table S2.**  **Criteria used to determine the optimal number of clusters when considering different numbers of clusters (from 2 to 6).**

| Number of clusters | average silhouette | Total Within Sum Of Squares | gap | Distance centroids |
| --- | --- | --- | --- | --- |
| 2 | 0.60 | 0.56 | 0.168 | 0.090 |
| 3 | 0.59 | 0.41 | 0.170 | 0.079 |
| 4 | 0.31 | 0.35 | 0.167 | 0.068 |
| 5 | 0.22 | 0.33 | 0.165 | 0.064 |
| 6 | 0.25 | 0.25 | 0.162 | 0.059 |

**Table S3. Performance of the k-prototypes algorithm for different number of clusters**

| k-prototypes |  |  |  |  |
| --- | --- | --- | --- | --- |
|  | average silhouette | Total Within Sum Of Squares | Calinski-Harabasz index | gap |
| 2 | 0.40 | 18.3 | 510.2 | 0.16 |
| 3 | 0.43 | 14.3 | 516.8 | 0.20 |
| 4 | 0.16 | 13.3 | 403.7 | 0.19 |
| 5 | 0.20 | 10.5 | 471.8 | 0.15 |
| 6 | 0.20 | 9.6 | 436.5 | 0.19 |
| 7 | 0.24 | 6.6 | 634.3 | 0.17 |

**Figure S2. Hierarchical clustering with Gower + Ward. The best partition to cut the dendrogram was based on the higher relative loss of inertia criteria**

**Table S4. Physical comorbidities across the three clusters**

| comorbidities | “low psychopathology” cluster  N=1075 (78.5%) | “affective” cluster  N=184  (13.4) | “Anxiety” Custer  N=110  (8.1) | Effect size, [CI] |
| --- | --- | --- | --- | --- |
| Heart disease | 502 (46.8) | 84 (45.7) | 55 (50.0) | 0.02 [0.00, 0.08] |
| Nervous disease | 79 (7.4) | 32 (17.4) | 12 (10.9) | 0.12 [0.06, 0.19] |
| Musculoskeletal disease | 503 (46.9) | 119 (64.7) | 74 (67.3) | 0.15 [0.10, 0.20] |
| Respiratory disease | 123 (11.5) | 26 (14.1) | 14 (12.7) | 0.03 [0.00, 0.09] |
| Stomach disease | 170 (15.8) | 39 (21.2) | 27 (24.5) | 0.07 [0.03, 0.14] |
| Genitourinary disease | 93 (8.7) | 20 (10.9) | 9 (8.2) | 0.03 [0.00, 0.09] |
| Endocrinological disease | 204 (19.0) | 40 (21.7) | 29 (26.4) | 0.05 [0.01, 0.11] |
| cancer disease | 75 (7.0) | 15 (8.1) | 6 (5.5) | 0.02 [0.00, 0.08] |
| Dermatological disease | 83 (7.7) | 21 (11.4) | 20 (18.2) | 0.10 [0.05, 0.17] |

**Table S5** “Comparison of baseline characteristics across the three centers”.

|  | Ferrara | Geneva | Hamburg | p-value |
| --- | --- | --- | --- | --- |
| Age, mean (SD) | 72.6 (5.3) | 73.2 (5.5) | 73.5 (5.0) | 0.06 |
| Sex, M, N (%) | 257 (59.1) | 247 (27.7) | 213 (28.2) | 0.09 |
| Number of school years, mean (SD) | 9.7 (4.8) | 14.3 (4.5) | 10.5 (2.4) | <0.01 |
| Number of school years group, N (%)  0-8  9-12  >13 | 248 (53.2)  64 (13.7)  154 (33.1) | 42 (8.3)  161 (31.6)  306 (60.1) | 66 (16.9)  244 (62.4)  81 (20.7) | <0.01 |
